# Residual lung lesions after completion of chemotherapy for gestational trophoblastic neoplasia: should we operate?

**DOI:** 10.1038/sj.bjc.6602899

**Published:** 2005-12-13

**Authors:** T Powles, P Savage, D Short, A Young, C Pappin, M J Seckl

**Affiliations:** 1 Department of Health Charing Cross Gestational Trophoblastic Disease Centre, Hammersmith Hospitals Campus of Imperial College London, Palace Rd, London W68RF, UK

**Keywords:** lung metastasis, chemotherapy, gestational trophoblastic disease

## Abstract

The significance of residual lung metastasis from malignant gestational trophoblastic neoplasm (GTN) after the completion of chemotherapy is unknown. We currently do not advocate resection of these masses. Here, we investigate the outcome of these patients. Patients with residual lung abnormalities after the completion of treatment for GTN were compared to those who had a complete radiological resolution of the disease. None of the residual masses post-treatment were surgically removed. In all, 76 patients were identified. Overall 53 (70%) patients had no radiological abnormality on CXR or CT after completion of treatment. Eight (11%) patients had residual disease on CXR alone 15 patients had residual disease on CT (19%). During follow-up, two patients (2.6%) relapsed. One of these had had a complete radiological response post-treatment whereas the other had residual disease on CT. Patients with residual lung lesions after completing treatment for GTN do not appear to have an increased chance of relapse compared to those with no residual abnormality. We continue to recommend that these patients do not require pulmonary surgery for these lesions.

Gestational trophoblastic neoplasia (GTN) encompasses a spectrum of disorders from the premalignant conditions of complete and partial hydatidiform moles (CHM or PHM) to the malignant disorders of invasive mole, choriocarcinoma, and placental site trophoblastic tumour (PSTT). About 16% of CHM and 0.5% of PHM transform into any of the malignant forms of GTN. Choriocarcinoma and PSTT can also develop after any type of pregnancy, including miscarriages and term deliveries (Seckl *et al*, 2000; Palmieri *et al*, 2005). Malignant GTN is usually diagnosed by a plateaued or rising human chorionic gonadotrophin (hCG) level some weeks following uterine molar evacuation. Histological confirmation is rarely performed because of the risk of life-threatening haemorrhage.

Malignant GTN can metastasis, most commonly to the lungs (Kumar *et al*, 1988). The vast majority of these patients are cured with chemotherapy despite the presence of metastatic disease, although those with liver and/or brain metastases have a poorer prognosis (Bower *et al*, 1997; Newlands *et al*, 2000). On completion of chemotherapy, most metastatic lesions will be completely resolved. However, in some patients, residual lesions persist usually in the lungs. The underlying concern is that such lesions may still harbour nests of active cancer, so should these lung lesions be removed?

For nongestational choriocarcinomas, which despite being pathologically similar are genetically and clinically distinct from GTN, the answer is yes. This is because residual masses may contain active cancer regardless of normal tumour markers (Thompson and Goldstein, 1969; Gels *et al*, 1997; Hartmann *et al*, 1997a, 1997b). However, for GTN, the situation is less clear as the disease is more chemosensitive and has an even lower relapse rate than that of nongestational choriocarcinomas arising from ovary (Sarwar *et al*, 2004). Therefore, our centre has historically observed residual GTN masses postchemotherapy with the exception of those in the brain, the latter being a site where chemotherapy might be less effective because of the blood–brain barrier (Newlands *et al*, 2002).

So is it safe to avoid resection of residual GTN lesions in the chest? Here, we attempt to address this issue by comparing the outcome for patients with or without residual radiological abnormalities in the chest, following chemotherapy for GTN.

## PATIENTS AND METHODS

The Charing Cross GTN database was screened to identify all women diagnosed between 1995 and 2004, with GTN and lung metastasis as the only site of metastasis. Patients with known metastases elsewhere including the liver and brain were excluded as these sites are associated with a worse prognosis and could confound the results. Only patients whose hCG levels had normalised for 6 weeks during chemotherapy and remained normal for 6 weeks following completion of chemotherapy, and were therefore biochemically thought to be cured, were included in the subsequent analysis. Clinical, biochemical and radiological details (either computer tomography (CT) or chest X-ray (CXR), or both) at presentation and on completion of chemotherapy were recorded. At presentation for chemotherapy, patients were staged according to nationally and internationally accepted criteria for GTN, stratified into low- or high-risk groups, and treated accordingly (Bagshawe, 1976; Kohorn, 2001; McNeish *et al*, 2002). Individuals with low-risk disease were treated with subcutaneous methotrexate and folinic acid, whereas those with high-risk disease were given weekly combination chemotherapy, comprising of etoposide, methotrexate, and actinomycin alternating with cyclophospohmide and vincristine (EMA/CO). Resistance to methotrexate occurred in a minority of patients while on treatment, and therapy was changed to either actinomycin or EMA/CO depending on the level of hCG at the time of resistance as previously described (McNeish *et al*, 2002). During follow-up, disease-free patients outcome was recorded and plotted according to the Kaplan–Meier method. This study was approved by our local institutional review board.

## RESULTS

Between 1995 and 2004, 681 patients received chemotherapy for malignant GTN. At presentation, 76 patients (11%) had lung metastases as the only overt metastatic site, and following chemotherapy, all had normal serum hCG levels and were thought to be cured. Nevertheless, 23/76 (30%) had persistent pulmonary radiological lesions. No pulmonary lesions were identified in the remaining 53 patients on either CXR and/or CT scanning after treatment.

The characteristics of patients with and without radiological abnormality, at the end of treatment are shown in Table 1. It is noteworthy that the median age at presentation, initial hCG, duration of treatment and proportion of patients receiving combination treatment, in the two groups are not significantly different (*P*>0.05 for each). Table 2 compares and contrasts specific factors including number and size of lung metastasis at presentation, the type of radiological investigation used at the end of treatment (CXR or CT), and the type of GTN with the risk of having persistent pulmonary lesions. Of these factors, only patients with lung lesions >2 cm at the start of the treatment or those who were imaged by CT scan (rather than CXR) were found to have a significantly increased chance of residual disease on completion of treatment (Table 2). In contrast, neither the number of metastases at presentation nor the presence of confirmed choriocarcinoma altered the risk of detecting residual lesions.

Overall, two patients relapsed after chemotherapy. Both patients had nonmolar choriocarcinoma that followed term delivery. One patient had multiple metastasis at the start of the treatment, the largest of which was 5 cm, and an hCG of 102 000 IU l^−1^. She was treated with combination chemotherapy for 21 weeks. At the end of the treatment, she had three residual masses on CT scanning ranging between 1 and 1.5 cm. Relapse occurred 3 months after the completion of treatment with a rising hCG. She went on to receive second line chemotherapy and eventually developed chemotherapy resistant disease and died from progressive GTN. The second patient had numerous 1–3 cm lung metastasis and an hCG of 36 000 IU l^−1^ at diagnosis. She received 17 weeks of combination chemotherapy, which resulted in complete resolution of the lung metastasis on CXR. The disease relapsed in multiple sites, including the brain, 6 months later. She also eventually died from her disease.

The 5-year disease-free outcome of the whole group was 97% (95% CI: 3–100%). For those patients with and without a residual abnormality, the 5-year disease-free survival was 95% (95% CI: 86–100%) and 98% (95% CI: 94–100%), respectively.

**Figure 1:**
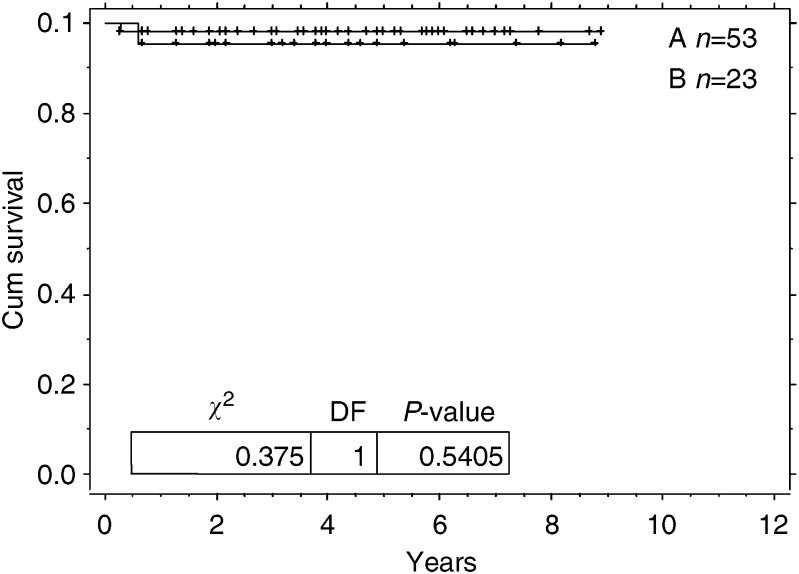
Kaplan–Meier graph for cumulative relapsed free survival. Arm A=no residual disease post-treatment. Arm B=residual disease post-treatment.

There was no significant difference in the disease-free outcome of the patients with or without persistent radiological abnormality (Figure 1).

## DISCUSSION

Little is known about the outcome of patients with residual pulmonary lesions, following successful chemotherapy for GTN with lung metastases at diagnosis. Additionally, there is uncertainty regarding the management of these residual lesions with some advocating surgical removal (Ilancheran, 1998). However, the work presented here shows that residual lung abnormalities in women who are in marker remission following chemotherapy for malignant GTN do not appear to have an increased risk of relapse. Consequently, we believe that these lesions simply represent dead tissue. Nevertheless, the absolute number of relapses in our study was small, so we cannot exclude the possibility that there may be a slight increased risk of relapse in patients with residual lung lesions when a larger cohort of patients has been studied. So is there any other data in the literature that might confirm or refute our findings?

There have been several smaller studies investigating the role of surgical resection for lung metastasis in GTN (Tomoda *et al*, 1980; Saitoh *et al*, 1983; Xu *et al*, 1985; Jones *et al*, 1993). However, unlike our study, all these have focused on surgery in patients with an elevated hCG associated with chemoresistant tumours and many were carried out before the introduction of curative combination chemotherapy for high-risk disease. The most recent of these studies was published in 1993 (Jones *et al*, 1993). Seven patients had surgical resection after chemotherapy; none had a normal serum hCG at the time of surgery. Additionally, five of the patients had extra pulmonary metastasis known to be associated with a poor prognosis. Indeed, three of these patients died of extrapulmonary disease. Interestingly, two patients with isolated pulmonary metastasis were cured by surgery as others have previously reported (Sink *et al*, 1981). Thus, a thoracotomy in the presence of a raised hCG is only likely to be helpful for isolated chemoresistant lesions and not when there is widespread disease (Sink *et al*, 1981). However, none of these studies really provides any additional data to that presented here concerning the management of residual lesions when the patients are in biochemical complete remission with a normal hCG.

In our study, not all the patients had the same radiological investigation at the end of treatment. Indeed, a higher proportion of patients with radiological abnormality at the end of treatment had CT scans. This is perhaps not surprising, as CT scanning is more sensitive at identifying lung lesions than CXR. Nevertheless, our results suggest that the observation of persistent radiological abnormalities at the end of the treatment has no bearing on the outcome for these patients. Therefore, the type of radiological test performed may be irrelevant. In view of the radiation exposure related to CT scanning, we do not recommend this investigation at the end of the treatment in this young patient population. Moreover, since radiological residual abnormalities in the chest do not appear to predict subsequent disease course, and relapses can be easily serologically detected by a rising hCG, one might be tempted to advocate no chest imaging at this time. Nevertheless, we still recommend performing a follow-up CXR on completion of therapy for women with lung metastases at presentation. This is because such imaging provides a useful baseline for future comparison. For example, should the patient undergo chest imaging for other reasons years later, the finding of a lung lesion might not necessarily prompt additional investigation if comparison with the previous films postchemotherapy shows no change. In addition, should the patient relapse and a CXR demonstrate a lung lesion, it would be important to know whether this was new, enlarged from before, or old and unchanged.

Perhaps unsurprisingly, this study also shows that patients with residual disease at the end of treatment have larger lung metastasis at diagnosis (Table 2). It may be that larger pulmonary metastasis result in more nonviable tissue after the completion of treatment.

In summary, chemotherapy alone cures the majority of patients with isolated lung metastasis. Radiological abnormalities at the end of treatment are of no prognostic significance if the patient is in hCG remission, and excision of these lesions does not therefore seem reasonable.

## Figures and Tables

**Table 1:** Comparability of patient groups with and without residual lung lesions following chemotherapy for malignant GTN associated with lung metastatses

	**All patients**	**No radiological abnormality after chemotherapy**	**Radiologic abnormality after chemotherapy**
Number	76	53	23
Median age at presentation	31 years (range: 17–61)	31 years (range: 17–61)	32 years (range: 18–58)
Median follow-up	5 years (range: 1–9)	4 years (range: 1–9)	5 years (range: 1–9)
hCG at start of treatment	109 00 IU l^−1^ (range 150–2 300 000 *μ*mol l^−1^)	110 000 IU l^−1^ (range 150–2 300 000 *μ*mol l^−1^)	106 000 IU l^−1^ (range 170–1 800 000 *μ*mol l^−1^)
Median duration of treatment	18 weeks (range 10–33)	18 weeks (range12–33)	16 weeks (range 10–29)
Proportion of high-risk patients (score>8)	48 (63%)	33 (62%)	15 (65%)
Number of relapses	2	1	1

**Table 2:** Influence of CXR verses CT, size of lesion, number of lesions and type of malignant GTN on risk of having a persistent pulmonary lesion after chemotherapy

**Radiological investigation at completion of chemotherapy**	**No radiological abnormality after chemotherapy**	**Radiological abnormality after chemotherapy**
CXR	34	8
CT	19	15
*χ*^2^ *P*=0.018		
		
Size of largest lung metastasis at the beginning of treatment	No radiological abnormality after chemotherapy	Radiological abnormality after chemotherapy
<2 cm	48	12
>2 cm	5	11
*χ*^2^ *P*=0.001		
		
Number of lung metastasis at the beginning of treatment	No radiological abnormality after chemotherapy	Radiological abnormality after chemotherapy
Less than 10	38	16
10 or more	15	7
*χ*^2^ *P*=0.85		
		
Type of GTN	No radiological abnormality after chemotherapy	Radiological abnormality after chemotherapy
Post molar malignant GTN (either invasive mole or choriocarcinoma)	34	13
Known choriocarcinoma	19	10
*χ*^2^ *P*=0.59		
